# Grating Interferometer with Redundant Design for Performing Wide-Range Displacement Measurements

**DOI:** 10.3390/s22103738

**Published:** 2022-05-14

**Authors:** Weinan Ye, Rong Cheng, Ming Zhang, Yu Zhu, Leijie Wang, Jinchun Hu, Xin Li

**Affiliations:** 1 State Key Laboratory of Tribology, Department of Mechanical Engineering, Tsinghua University, Beijing 100084, China; yewn@tsinghua.edu.cn (W.Y.); chengr@mail.tsinghua.edu.cn (R.C.); zhangm@tsinghua.edu.cn (M.Z.); zhuyu@tsinghua.edu.cn (Y.Z.); hujinchun@tsinghua.edu.cn (J.H.); lixin_09@mail.tsinghua.edu.cn (X.L.); 2 Beijing Lab of Precision/Ultra-Precision Manufacture Equipment and Control, Tsinghua University, Beijing 100084, China

**Keywords:** grating interferometer, wide-range, displacement measurement, six degree-of-freedom

## Abstract

Grating interferometers that use large two-dimensional grating splice modules for performing wide-range measurements have significant advantages for identifying the position of the wafer stage. However, the manufacturing process of large two-dimensional grating splice modules is very difficult. In contrast to existing redundant designs in the grating line dimension, we propose a novel interferometric reading head with a redundant design for obtaining wide-range displacement measurements. This interferometric reading head uses a one-dimensional grating splice module, and it was observed to be compatible with two orthogonal gratings. We designed a grating interferometer system composed of four reading heads to achieve a wide range of measurements and verified it using ZEMAX simulation. By conducting experiments, we were able to verify the compatibility of the reading head with gratings possessing different grating line directions; the measurement noise was found to be less than 0.3 nm.

## 1. Introduction

Grating interferometers are widely applied in the displacement measurement of six degree-of-freedom (6-DOF) planar motions (large-stroke translational motion in a plane, small-stroke vertical, and rotational motion) [1], such as in photolithography scanners and coordinate measuring machines [2,3,4,5], due to their characteristics of noncontact measurement, high resolution, and high reproducibility. As a variant of the laser interferometer, the grating interferometer uses a Doppler effect of diffraction and reflection to detect the displacement in the grating vector direction and the normal direction. Instead of lengthening the optical path as in a laser interferometer, a grating interferometer increases the measurement range of the grating vector direction using large grating sizes without changing the optical path [6,7,8]. In addition to size, the dimension of the grating (one-dimensional line (1-D) or two-dimensional grid (2-D)) is critical to the measurement range of a reading head [9].

**Figure 1:**
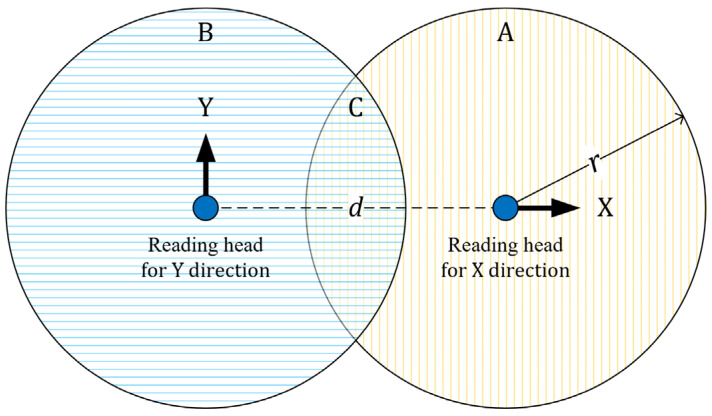
Requirements of a grating interferometer system for grating size and line dimension; *d* denotes the distance between two reading heads; *r* is the radius of the circular measurement range of the reading head; A is the grating area for X direction measurement; B is the grating area for Y direction measurement; C is the grating area for X and Y direction measurements.

To achieve 6-DOF displacement measurement by the combination of translational sensors, it is necessary to arrange at least six measuring axes in at least three positions that are not collinear [10]. The measurement range of the grating interferometer with a single reading head is determined by the grating’s size; however, the measurement range of the grating interferometer system composed of multiple reading heads is limited by the distances between two reading heads [11]. Consider a system as shown in Figure 1, wherein two reading heads measuring different directions are installed on the measured object with a distance *d* between the reading heads. If the motion range, 2*r*, of the measured object is greater than *d*, there must exist an area in the grating that can work with the two reading heads. The solution, which was provided by ASML (the leading manufacturer of photolithography scanners in the world), is 2-D gratings. The grating interferometer system used in the immersion photolithography scanners of the company uses a module composed of several large 2-D gratings to identify the full stroke position of the wafer stage [12]. However, these large 2-D gratings are extremely difficult and expensive to manufacture [13].

To overcome these issues, a novel interferometric reading head with a redundant design has been proposed in this study for attaining wide-range displacement measurements. This reading head had two measurement degrees of freedom (DOF): the grating vector and normal directions. In addition to a pair of measuring beams similar to that used in the conventional design, another pair of redundant measuring beams was added to extend the measurement range. The two pairs of measuring beams could produce a Doppler effect on two gratings with orthogonal grating lines, sharing the reference beams, multimode fibers transmitting the interference signals, and electronics board for signal processing.

## 2. Interferometric Reading Head with Redundant Design

**Figure 2:**
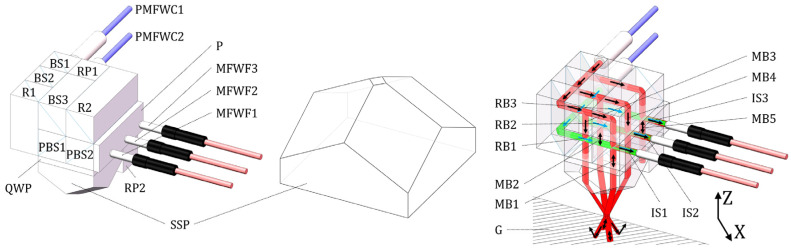
Structure of the novel grating interferometer with the redundant design: PMFWC denotes the polarization-maintaining single-mode fiber with collimator; MFWF is the multimode fiber with a ferrule end; BS is the beam splitter; R is the reflector; RP is the rectangular prism; PBS is the polarizing beam splitter; QWP is the quarter-wave plate; SSP is the special-shaped prism; P is the polarizer; MB is the measuring beam; RB is the reference beam; IS is the interference signal; G is the grating with the lines on the surface representing the direction of the grating lines.

Figure 2 shows the novel grating interferometer with a redundant design. The proposed grating interferometer, based on the spatial heterodyne interference principle, used two polarization-maintaining single-mode fibers with collimators to transmit dual-frequency lasers. The measuring beams with frequency f1, indicated by the black arrow and the reference beam with frequency f2, indicated by the blue arrow, were split into three parallel beams by BS1, BS2, and R1. Three p-polarized reference beams, RB1 to RB3, were transmitted through PBS1 and PBS2 and were subsequently directly coupled into the multimode fibers through the ferrule ends. Five p-polarized parallel measuring beams, MB1 to MB5, of which three were split by BS3 and R2, were transmitted through PBS1, PBS2, and QWP. After undergoing reflection by RP2 and PBS2, MB5 was coupled into multimode fiber, forming an interference signal, IS3, with RB3, which was used to compensate for the polarization-maintaining fiber transmission and thermal drift errors. After undergoing refraction by SSP, MB1 and MB2 were diffracted (first order) by the grating (the direction of the grating lines is shown in the figure) and returned along their original paths, producing the Doppler effect and, thus, obtaining relative displacement information between the reading head and grating. After undergoing reflection by PBS1 and PBS2, MB1 and MB2, which are the ±first-order light output by one-time diffraction, formed two interference signals, IS1 and IS2, with RB1 and RB2. When only translational displacement is considered, the Doppler phase shift *φ* of two interference signals could thereafter be obtained according to Equation (1) [2], and the relative displacement of *Dx* and *Dz* in X and Z directions between the encoder and grating can be calculated according to Equations (2) and (3). As a pair of redundant measuring beams, MB3 and MB4 no longer returned to the reading head after being reflected on the grating but can diffract on the grating orthogonal to the illustrated grating:

(1) $$\varphi =-2\pi \left(\frac{m}{p}Dx+\frac{n\left(\cos\theta_{m}+\cos\theta_{inc}\right)}{\lambda }Dz\right)$$

(2) $$Dx=\frac{p\left(\varphi_{1}-\varphi_{2}\right)}{4\pi }$$

(3) $$Dz=\frac{\lambda \left(\varphi_{1}+\varphi_{2}-2\varphi_{3}\right)}{8\pi \cos\theta }$$

where *p* denotes the grating pitch, *m* is the diffraction order, *λ* is the wavelength of the laser, *n* is the refractive index of the air, *θ_m_* and *θ_inc_* are the polar angle of the *m*-order diffracted beam and the incident beam, respectively, both of which are equal to *θ* in the proposed reading head, and *φ*_1_, *φ*_2_, and *φ*_3_ are the phase shifts of IS1, IS2, and IS3, respectively.

**Figure 3:**
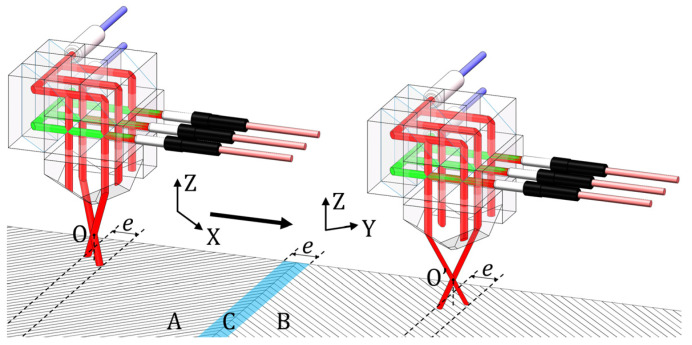
Working states of the reading head with different gratings.

As shown in Figure 3, the parts of the measuring beams that were not diffracted were hidden to indicate the working states of the reading head on different gratings. When the reading head moved from the above grating A to above grating B (O to O′), the measurement DOF changed from X and Z to Y and Z. During this movement, the reading head was unable to operate in area C, for which its width was equal to the length, *e*, covered by the two measuring beams on the grating surface. In essence, the grating interferometer, composed of the reading head with a redundant design and a 1-D grating splice module, had three working states: the X and Z available state, unavailable state, and Y and Z available state.

## 3. Grating Interferometer System for Wide Range of 6-DOF Displacement Measurements

**Figure 4:**
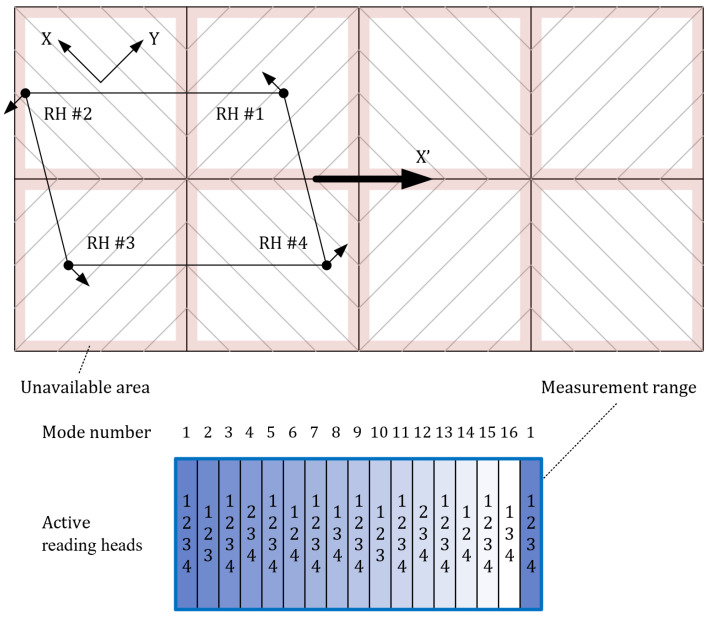
Grating interferometer system with 16 different working modes to achieve a wide range of 6-DOF displacement measurements. The reading heads (RH) are simplified as dots, and their measurement directions on the grating plane are indicated by arrows. Red indicates areas where the reading heads were unavailable. The blue bold lines indicate the measurement range divided into 17 areas.

Due to the three working states of a single reading head, a system composed of multiple reading heads would inevitably have multiple working modes. In this study, a grating interferometer system consisting of four proposed 2-DOF (X/Y and Z) reading heads and eight 1-D gratings was designed to demonstrate the mechanism through which a wide range of 6-DOF displacement measurements could be achieved by switching between multiple modes. As shown in Figure 4, the four reading heads were arranged in a parallelogram shape on the motion stage such that any two adjacent gratings in two rows had different grating line directions. During the full stroke of the motion stage from left to right, the measuring system switched to 16 different working modes and finally returned to the original working mode. The measurement directions of the four reading heads in the different modes are listed in Table 1. When reading head #*i* is displaced in 6-DOF relative to the grating, the nonlinear relationship between its readings and the displacement can be expressed as follows:

(4) $$\begin{array}{ccc} \varphi_{ij}-\varphi_{i3}=f\left(Dx^{\prime },Dy^{\prime },Dz^{\prime },Rx^{\prime },Ry^{\prime },Rz^{\prime }\right) & i=1,2,3,4 & j=1,2 \end{array}$$

where *φ_ij_* denotes the phase shifts of IS1 and IS2 of reading head #*i*, *φ_i3_* is the phase shift of IS3, and (*Dx′*, *Dy′*, *Dz′*, *Rx′*, *Ry′*, *Rz′*) is the 6-DOF displacement of the motion stage. Using the 9 or 12 readings, an even-determined (three-active reading head mode) or over-determined (four-active reading head mode) equation system can be established, and the 6-DOF displacement can be obtained by solving the equation system. According to our previous research, the sixteen working modes, which included eight four-active reading head modes and eight three-active reading head modes, used the same method to calculate 6-DOF displacements; however, their coefficient matrices were different [14,15]. In some applications with very small rotation ranges or low measurement accuracy, the equation system can be linearized using a Taylor series expansion, and its solution is similar to the displacement calculation method in [1].

In the measurement range of the grating interferometer system, the four-active reading head areas were only adjacent to the three-active reading head areas and vice versa. Thus, when the motion stage moved from a four-active reading head area to an adjacent three-active reading head area, one reading head would stop working and the system would switch to the corresponding coefficient matrix for the displacement calculation. When the motion stage moved from a three-active reading head area to an adjacent four-active reading head area, in addition to switching to the corresponding coefficient matrix, the initial phase of the reading head to be activated would be calculated using the 6-DOF displacements measured by the three active reading heads since the sensors were incremental.

**Figure 5:**
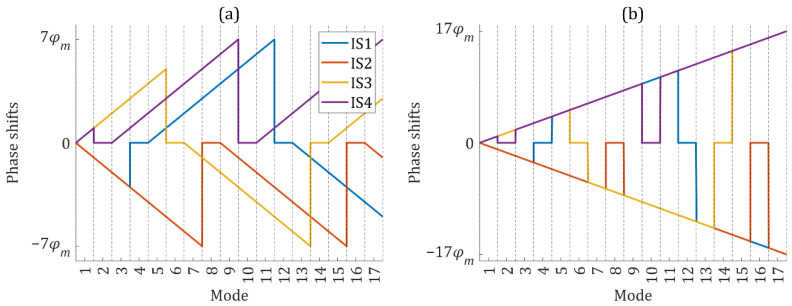
(**a**) Phase shifts of the IS1 of the four reading heads obtained by simulation. (**b**) Phase shifts after compensating for the initial phases.

To verify the compatibility of the reading head with gratings of different grating line directions and the continuity between multiple working modes, the simulation model of the described grating interferometer system was constructed in the nonsequential mode of ZEMAX [16]. The sizes of the 17 measurement subranges divided by the working modes were the same. Figure 5a shows the phase shifts of the IS1 of the four reading heads obtained by simulation as the motion stage moved uniformly in the X′ direction with one DOF. The phase shifts of the IS2 were negative for IS1 in this single DOF motion. The phase shifts of IS3 were zero in the simulation without polarization-maintaining fiber transmission and thermal drift errors. Upon entering the unavailable area, the reading head stopped working and the phase shift became zero. When it was out of the unavailable area, the reading head restarted, but the measurement direction changed. As shown in Figure 5b, the phase shifts after compensating for the initial phases did not change from zero when the reading head was out of the unavailable area but conformed to the continuous linear change of uniform motion. This indicates that the calculated 6-DOF displacements of the different working modes were consistent with the given uniform motion.

## 4. Experiments

**Figure 6:**
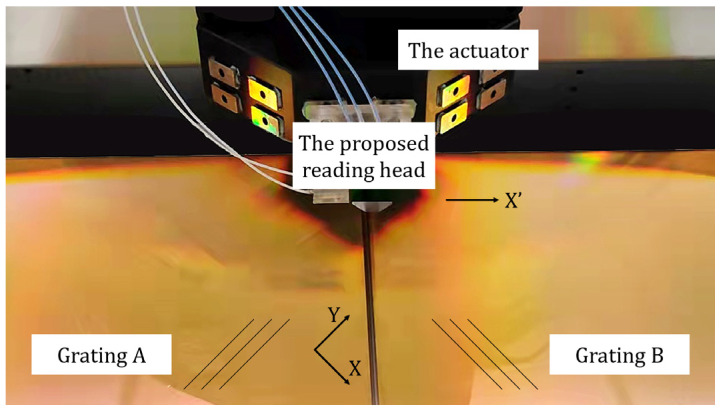
Experimental device. Two groups of parallel lines indicate different grating line directions.

In addition to the simulation, an experimental device, as shown in Figure 6, was set up to verify the compatibility of the reading head with gratings possessing different grating line directions. The proposed reading head was mounted on the actuator of a commercial linear motion stage. Two customized rectangular 1-D gratings (170 mm × 170 mm) with a pitch of 833.3 nm, possessing orthogonal grating lines at an angle of 45° with the edge, were arranged next to each other on the stator. There were no grating lines within 1 mm of the grating edge, and the gap between the two gratings was 0.2 mm wide. In addition, the grating interferometer system used a self-developed dual-frequency (the heterodyne frequency is 20 MHz) laser source with a wavelength of 780 nm and a ZYGO electronics board.

**Figure 7:**
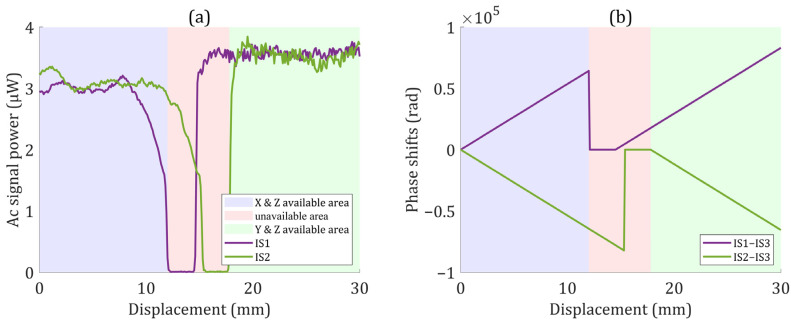
(**a**) AC signal power measured by the electronics board. (**b**) Phase shifts. The abscissa axis was converted to the displacement by multiplying with the ideal velocity. IS1-IS3 and IS2-IS3 denote phase shifts after correcting the polarization-maintaining fiber transmission and thermal drift errors.

Figure 7 shows the phase shifts and AC signal power measured by the electronics board when the motion stage moved 30 mm along the X′ direction at a speed of 10 mm/s. According to the measurement results of the phase shift, the measurement range could be divided into three areas: the X and Z available areas, unavailable areas, and Y and Z available areas. The areas where only one measurement axis was available, where both measurement axes were unavailable, and where the two measurement axes worked with different gratings are defined as unavailable areas.

**Figure 8:**
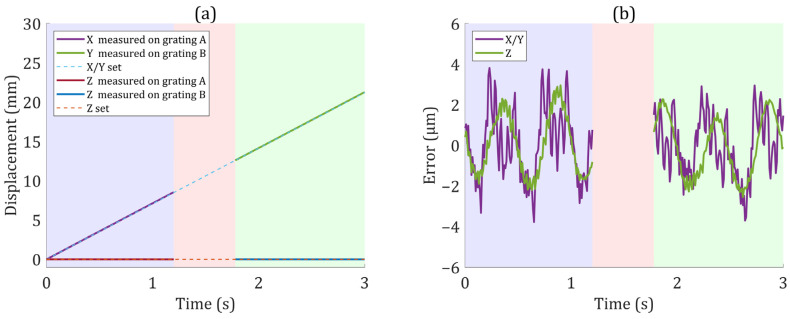
(**a**) Displacements measured by the grating interferometer. The displacement set in both X and Y directions was $\sqrt{2}/2$ of the displacement in the X′ direction because the measurement direction was at a 45° angle with the motion direction. (**b**) Motion errors of the actuator after correcting linear errors such as cosine errors.

After modifying the initial phases, the displacements measured by the grating interferometer, which were consistent with the actual motions, are shown in Figure 8a. Based on measurement results, the motion errors of the actuator after correcting for linear errors such as cosine errors are shown in Figure 8b. A motion accuracy of 5 μm was obtained, which was in agreement with the performance of the linear motion stage.

**Figure 9:**
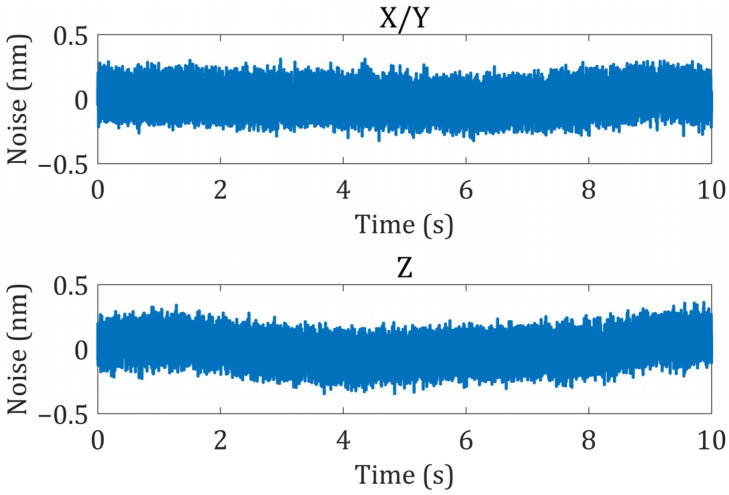
Measurement noise of the proposed grating interferometer.

To test the measurement noise of the proposed grating interferometer, a rigid structure was machined to hold the grating and reading head together in terms of the relative stiffness. The device was placed on a high-performance, vibration-isolation platform. As shown in Figure 9, the noise in the X or Y direction obtained from the test was 0.25 nm (3σ), whereas that obtained in the Z direction was 0.27 nm (3σ). After analysis in the frequency domain, the measurement noise mainly consists of the white noise of the electronics board and low-frequency drift. In another study, we confirmed that low-frequency drift comes from multimode fibers disturbed by the environment.

## 5. Conclusions

A novel grating interferometer with a redundant design for achieving wide-range displacement measurements was proposed in this study, which helps in reducing the difficulty and cost of application. We found that the proposed interferometric reading head, which uses a 1-D grating splice module, was compatible with two orthogonal gratings. To achieve a wide range of measurements, a grating interferometer system composed of four reading heads was designed, and its performance was verified using ZEMAX simulation. Furthermore, the results revealed that the reading head was compatible with gratings of different grating line directions, and the measurement noise was found to be less than 0.3 nm. Future studies may be aimed at improving the measurement noise and rotation range of the proposed grating interferometer.

## Figures and Tables

**Table 1:** Measurement directions of the four reading heads in different modes.

Reading Head	Working Modes															
	1	2	3	4	5	6	7	8	9	10	11	12	13	14	15	16
1	X	X	X	\	Y	Y	Y	Y	Y	Y	Y	\	X	X	X	X
2	Y	Y	Y	Y	Y	Y	Y	\	X	X	X	X	X	X	X	\
3	X	X	X	X	X	\	Y	Y	Y	Y	Y	Y	Y	\	X	X
4	Y	\	X	X	X	X	X	X	X	\	Y	Y	Y	Y	Y	Y
